# Real-Time Assessments of the Strength of Program Implementation for Community Case Management of Childhood Illness: Validation of a Mobile Phone-Based Method in Malawi

**DOI:** 10.4269/ajtmh.14-0396

**Published:** 2015-03-04

**Authors:** Elizabeth Hazel, Agbessi Amouzou, Lois Park, Benjamin Banda, Tiyese Chimuna, Tanya Guenther, Humphreys Nsona, Cesar G. Victora, Jennifer Bryce

**Affiliations:** Johns Hopkins Bloomberg School of Public Health, Baltimore, Maryland; United Nations Children's Funds, New York, New York; National Statistical Office, Zomba, Malawi; Save the Children, Lilongwe, Malawi; Save the Children, Washington, DC; Ministry of Health, CHSU, IMCI Unit, Lilongwe, Malawi; Universidade Federal de Pelotas, Pelotas, Brazil

## Abstract

Health surveillance assistants (HSAs) in Malawi have provided community case management (CCM) since 2008; however, program monitoring remains challenging. Mobile technology holds the potential to improve data, but rigorous assessments are few. This study tested the validity of collecting CCM implementation strength indicators through mobile phone interviews with HSAs. This validation study compared mobile phone interviews with information obtained through inspection visits. Sensitivity and specificity were measured to determine validity. Using mobile phones to interview HSAs on CCM implementation strength indicators produces accurate information. For deployment, training, and medicine stocks, the specificity and sensitivity of the results were excellent (> 90%). The sensitivity and specificity of this method for drug stock-outs, supervision, and mentoring were lower but with a few exceptions, still above 80%. This study provided a rigorous assessment of the accuracy of implementation strength data collected through mobile technologies and is an important step forward for evaluation of public health programs.

## Introduction

As the 2015 deadline for achieving Millennium Development Goals 4 and 5 approaches, maternal, newborn, and child health programs are being scaled up in low- and middle-income countries (LMICs). Global awareness of the importance of program accountability has increased, pushed by the 2010 Commission on Information and Accountability (CoIA) for Women's and Children's Health convened by the United Nations Director General. Commissioners called for governments and partners to provide “better information for better results” taking advantage of innovative information and communication technologies to improve the quality and speed of data collection.^1^

Mobile phones are a burgeoning technology in LMICs. Mobile cellular subscriptions in Africa have increased from 87 million in 2005 to 545 million in 2013.^2^ Mobile health (mHealth) is a growing field that uses mobile technology to provide health services, access patient data, track disease, and support health information systems (HISs).^3^ This technology holds potential to improve the quality, timeliness, and availability of data on health programs in LMICs, but there are few rigorous assessments of their use. mHealth pilot projects to date have not been fully integrated into the HISs and have been largely developed to respond to the needs of projects rather than Ministries of Health (MOHs).^4^

Malawi has been implementing community case management (CCM) of major childhood illnesses since 2008 through trained health workers known as health surveillance assistants (HSAs). The MOH and its partners have agreed on a set of CCM program delivery indicators known as implementation strength indicators, and they are using them as the basis for program monitoring. Implementation strength refers to the quantity of the program strategy that is delivered at the population level and includes process and output information as outlined in the evaluation framework.^5^ The indicators focus on HSA deployment, training, use of services, medicine/supply stocks, and supervision. However, the routine monitoring systems for CCM are still being scaled up to generate information that is sufficiently accurate to guide program decision-making. The MOH requested assistance in assessing whether mobile phones represented a feasible option for collecting accurate, real-time data on CCM implementation strength that could be used to strengthen CCM programs at district levels and below.

Several African countries have piloted the use of mHealth technologies to collect routine health reporting data from community health workers (CHWs) supporting the HISs.^6^–^8^ However, there is an urgent need for more rigorous assessments of the potential role.^3^ This study aims to test the validity of CCM implementation strength data collected through mobile phone interviews with HSAs. The main objectives are to determine (1) whether mobile phone interviews with HSAs produce accurate and reliable data on CCM implementation strength and (2) the financial cost of the method.

## Methods

This study was designed to validate CCM implementation strength data collected through mobile phone interviews with HSAs by comparing HSA responses with information obtained through inspection visits to the health center to review supervision/monitoring records and the village clinics to observe medicine/supply stocks and the CCM sick child register. Inspection visits were considered the gold standard.

We conducted the study in two purposefully selected districts of Malawi: Dowa and Ntcheu. These districts were selected, because both had implemented the new government forms that included selected CCM implementation strength indictors for at least 6 months, had sufficient numbers of CCM-trained HSAs and health centers to meet the sample size requirements, and were a close distance from the capital city to facilitate supervision and minimize transport costs.

The District Health Management Teams (DHMTs) in the two districts were asked to provide a list of all of the HSAs trained in CCM and their corresponding health centers. A sample size of at least 100 observations per method was required to validate the method by estimating its sensitivity and specificity with a margin of precision (equal to two SEs) of ±10% points. A previous pilot study found that 40% of the randomly selected HSAs sampled in Balaka District were inaccessible by mobile phone because of network coverage issues.^9^ Assuming an additional 10% non-response, a simple random sample of 250 CCM-trained HSAs was selected to ensure that at least 100 HSAs would be able to be interviewed by mobile phone.

The health center was the unit of selection. We estimated the number of health centers to generate 250 HSAs for the study with roughly an equal number in each district. With this information, we randomly selected health centers in Dowa and included all health centers in Ntcheu (the latter having much fewer health centers). From each chosen health center, we randomly selected seven CCM-trained HSAs. If fewer than seven HSAs were working at that health center, we selected all HSAs. This method resulted in 244 CCM-trained HSAs; six additional HSAs were randomly selected from Dowa District to meet the 250 HSAs goal.

A selected HSA was excluded from the study sample if the HSA (1) had not been trained in CCM, (2) was no longer working, or (3) did not consent to participate in the study. We found several errors in the district lists of HSAs trained in CCM. Some selected HSAs were no longer working or had moved to other health centers or districts. In these cases, we randomly selected a replacement HSA from the same health center. If no other HSAs were available to select, we randomly selected one from another health center.

The DHMTs were informed about study activities in advance and asked to provide the mobile phone numbers of the selected HSAs and supervisors. The interviewer teams began at the selected HSA's assigned health center, where they interviewed the HSA's supervisors and inspected routine monitoring reports for the previous 6 months. This information was used to assess the accuracy of responses provided by HSAs by mobile phone interviews for retrospective events: supervision, mentoring, and medicine stock-outs. The interviewers began at the health center to minimize risk of a supervisor observing specific HSA responses and protect confidentiality.

We then attempted to contact the HSAs by mobile phone. If we were unable to reach the HSA by phone after four attempts, the HSA was assigned to an alternative group to be interviewed face to face at a health center or their village clinic. After the HSA interview, the survey teams visited the village clinic of the interviewed HSA to inspect the medicine/supply stocks and the CCM register (Table 1). Follow-up visits were scheduled within 3 days of the phone interview. Finally, the interviewers administered a short questionnaire immediately after the inspection to determine whether there were any differences in the data reported by the HSA versus the gold standard. If discrepancies were found, the interviewer asked the HSA to determine the cause. Interviewers were not masked (blinded); one interviewer completed all three tools for each HSA.

Data collection took place from October to November of 2012. Data were collected on paper forms and double-entered using CSPro 5.0 (United States Census Bureau, Washington, DC).^10^ Stata, version 11 (Stata Corp LP, College Station, TX) was used for data cleaning and analysis.^11^ We calculated sampling weights using the ratio of HSAs selected and interviewed by mobile phone to all CCM-trained HSAs in each health facility. Each facility was treated as a cluster in the survey database, and clustering was incorporated in all analyses. The analysis focuses on the sensitivity and specificity of responses obtained from the mobile phone interviews compared with the inspection visits. We established 80% as the criterion for considering the results produced by the mobile phone method as adequate for the purposes of program monitoring and evaluation. For the mean number of children treated in the previous 7 days, we used a Bland–Altman test of mean differences to assess any differences between reported and observed data for each indicator.^12^ Details of the validation methods used for each implementation strength indicator are presented in Table 1.

The financial costs of conducting the mobile phone interviews were assessed by recording the total airtime allocated to interview staff. The airtime provided covered the consent process, the interview, and any telephone calls to organize visits and make appointments. We also estimated the airtime cost per completed mobile phone interview. Staff costs were not included in the assessment.

We received approval for these activities from the Johns Hopkins Bloomberg School of Public Health Institutional Review Board and the Ministry of Health, Malawi. All participants consented to the study procedures before data collection. To provide fully informed consent, we explained to HSAs before administering the mobile phone interview that a follow-up inspection visit would be conducted at their village clinic and assigned health center.

## Materials

Three types of questionnaires were used to collect data. The first was administered to the has by mobile phone. A second questionnaire was used to record the information collected through the inspection visits at the village clinic and health center. A third tool provided semistructured guidance for interviewing HSAs to explain any discrepancies found between the two methods. Interviewers from the Malawi National Statistics Office with survey experience participated in a 1-week training on study procedures and tools.

## Results

**Figure 1.**
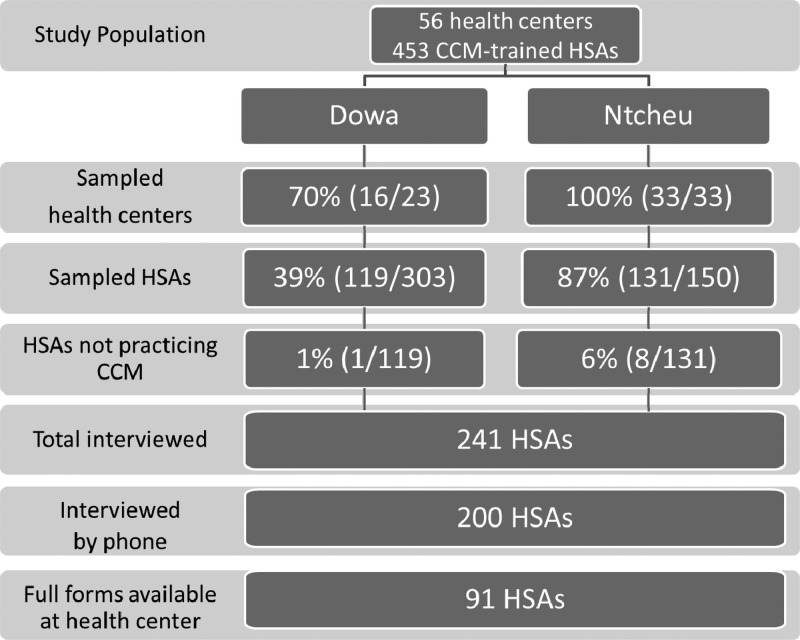
Health centers and the CCM-trained HSA population, selection and interviewed/data collected.

Among 250 CCM-trained HSAs randomly selected to participate in the study, 241 (96%) were available for interview, and of those (Figure 1), 200 (83%) HSAs were reached by mobile phone for the interview. The remaining 41 (17%) HSAs were interviewed face to face at the health center or their village clinic. We report here on 200 randomly selected CCM-trained HSAs interviewed by mobile phone. Complete sets of forms for the past 6 months were available at the health center for only 46% (91 of 200) of HSAs. For those indicators requiring validation through routine forms, we excluded those HSAs with incomplete forms from the analysis.

Table 2 shows the implementation strength results according to the two methods as reported by the HSAs interviewed by phone and as determined through the gold standard inspection. All results are weighted as described above, but unweighted results were virtually identical. Most (89%; 178 of 200) had seen at least one sick child in the 7 days before the interview, indicating that they were actively providing CCM services. Mobile phone reporting showed high levels of sensitivity and specificity for these indicators.

The observation results indicate that the frequency of supervision and mentoring was low, with only about one-third of the HSAs reporting receiving any supervision (30%; 60 of 200) or mentoring (29%; 57 of 200) in the previous 3 months. Sensitivity and specificity of the responses provided through mobile phone interviews for supervision/mentoring indicators were above 80%.

The percentage of HSAs with current stocks was at or above 80% for CCM medicines, and over one-half (60%; 118 of 198) of the HSAs had all key CCM medicines in stock on the day of the interview. Only those HSAs with complete sets of forms at the health centers were analyzed for stock-outs occurring in the previous 3 months. More than one-half (58%; 52 of 89) had not experienced a stock-out for any key CCM medicines in the previous 3 months.

Over three-fourths (83%; 165 of 199) of the HSAs reported having the minimum stocks of lumefantrine/artemether (LA; 2 × 6 packet) and cotrimoxazole (83%; 165 of 199). Reported minimum stocks of oral rehydration salt (ORS) solution (69%; 138 of 199) and zinc (67%; 133 of 199) were lower. Less than one-half of the HSAs (49%; 97 of 199) reported having minimum stocks for all key CCM medicines. Almost all (> 95% for all supplies) of the HSAs reported having all basic CCM supplies. The sensitivity and specificity of the mobile phone interview method were adequate (> 80%) for medicine stock-outs. However, the specificity for LA stock-out reporting was somewhat lower, indicating that some HSAs may be underreporting LA outages.

We investigated the reporting discrepancies for four indicators with the lowest levels of sensitivity and specificity. For the number of children treated in the previous 7 days, 83% of the discrepancies consisted of HSA reporting errors: errors in counting, failing to consult the register during the interview, and mishearing the question. The remaining discrepancies were attributed to interviewer counting errors. Over one-half of the errors in medicine stocks for the previous 3 months were attributed to an HSA mistake in reporting, such as misunderstanding the question, not calculating the 3-month period correctly, or forgetting stock-outs. One-third of the errors were caused by mistakes made on the HSA reporting forms that were used for the validation.

For supervision in the previous 3 months, one-third of the errors resulted from mistakes on the recording form used for the validation process. In some cases, the supervisor incorrectly reported the supervision according to the HSA reports. Interviewers were unable to determine the reason for the discrepancy for approximately one-quarter of the errors (28%). For mentoring, the interviewers were unable to determine a reason for the discrepancy in one-half of the cases (53%), whereas the remaining one-half of the cases were attributed to HSA reporting errors on the routine forms.

We compared the CCM implementation strength indicators among 41 HSAs interviewed face to face and 200 HSAs interviewed by mobile phone.^13^ Differences between the two groups were small. Generally, HSAs working in more remote areas had slightly fewer CCM medicines at their clinic at the time of the study (excluding LA), were somewhat less likely to be supervised and mentored, and were somewhat less likely to have treated a child in the previous 7 days than those working in network-connected areas (data not shown). For stock-outs, the sample was further restricted to the 45% (91 of 200) of HSAs whose records were complete. When comparing implementation strength indicators among those HSAs working at health centers with complete records for the previous 6 months and those working at health centers with incomplete records, differences were again small. The only noticeable differences regarded higher rates of supervision/mentoring and current stocks of zinc and cotrimoxazole in those with complete records.^13^

The estimated cost of the method (excluding the costs of validation) was 1,995 Malawian Kwacha (MWK) or 7 US dollars (USDs) per completed mobile phone interview (exchange rate for October 1, 2012).^14^ There was a slight variation in the airtime costs per interview between Dowa (6 USD per completed interview) and Ntcheu (7 USD per completed interview), which indicates that there may be geographic variation in the costs of the application of this method.

## Discussion

Using mobile phones to interview HSAs on CCM implementation strength produces accurate information. For HSA deployment and training and current and minimum medicine stocks and use, the results produced by the mobile phone method were excellent. The sensitivity and specificity of this method for medicine stock-outs, supervision, and mentoring were lower but with a few exceptions, still above 80%. Many of these discrepancies were reportedly caused by errors on the monitoring forms or HSA reporting errors during the interviews. Although almost all of the HSAs had basic CCM supplies in stock, the specificity of the phone interview method was lower for supplies than for medicines. HSAs reported having these supplies, but they were not actually present at the village clinic, particularly the sick child recording form. This form is used as a job aid for the HSAs; it may not be used frequently at the village clinic after HSAs become more experienced in managing sick children. The cost per interview of the mobile phone methodology is considerably less than what it would cost to conduct in-person monitoring visits to HSAs.

There are several limitations associated with this study. We selected districts close to the capital city and more accessible to national program staff; these findings might be less generalizable to more remote districts. However, our objective was not to provide representative information on implementation strength for the whole country but validate a new method. For such a purpose, national representativeness is not essential, because we have carried out an internal comparison of two data sources within the selected districts.

In the consent process, before the interview, the survey teams informed the HSAs that an inspection visit would occur at their village clinic to check their responses. This complied with international standards for human subjects research. HSAs may have given more accurate responses than they would under normal circumstances as a result. Follow-up visits occurred within 3 days of the initial phone interview, and it is possible that HSAs could have made changes to the register or drug/supply stocks to appear to have reported more accurately.

We asked all HSAs with reporting discrepancies to explain any discrepancies. To protect the confidentiality of the HSAs, we could not return to the health center for additional investigation. There could be bias with how the HSAs responded if they were concerned about appearing to have reported incorrectly or made mistakes. The qualitative data for reporting discrepancies should be interpreted with caution. The analysis was based on 83% (200 of 241) of all sampled HSAs, namely those who were reachable by mobile phone. This proportion was higher than what had been estimated before the survey, indicating that mobile network coverage is expanding in Malawi. For stock-outs, only 43% (91 of 200) had complete records at the health facility, and therefore, we further restricted the sample to those 91 HSAs. In both instances, bias is unlikely to have occurred because of the small magnitude of the differences among those included and not included in the analyses.

Our gold standard for indicators of supervision and stock-outs was based on inspection of monitoring records at the health centers, which were incomplete and may have data quality issues. However, we found good levels of sensitivity and specificity for the mobile phone method, regardless of potential issues with the routine reporting.

There are very few published studies systematically evaluating the accuracy of health information collected through mHealth.^3^ Several studies in Malawi have shown that mobile phones have the capacity to increase communication with distant communities and strengthen health programs.^15^,^16^

This paper presents one of the few assessments of the accuracy of program implementation strength data collected through mHealth technologies. Although more evaluations of mHealth programs are being published, there is still a lack of effectiveness of large-scale mHealth programs.^17^ Additional assessments will be needed as a basis for proposing that mHealth approaches be implemented at scale.^4^

Based on the results of this validation study, we recommend that Malawi conduct periodic mobile telephone interviews with front-line health workers as one component of their strategy for monitoring and evaluating the CCM program. This method holds particular promise in settings where the routine monitoring system for community-based programs is not yet fully functional. However, Malawi has reasonably high mobile phone network coverage compared with many sub-Saharan African countries. This method will not be useful in settings where connectivity is an issue.

This method for assessing the strength of program implementation also represents an important step forward for the rigorous evaluation of public health programs at scale. The low cost means that data can be collected from high proportions of health workers—perhaps even a census in some settings—and used to develop accurate snapshots of implementation strength for small geographic areas, such as districts. These snapshots and trend data produced through repeated applications of the method will provide program managers and implementers with the information needed to improve programs in real time. They will also provide program evaluators with a rich source of time series data that can be used to evaluate the causal relationships between specific program delivery strategies and population health outcomes and impact.^18^

Previous studies have noticed that mHealth solutions tend to be top down, with no built in feedback loop to CHWs.^19^ Solutions to improve data use for paper-based reporting systems have shown that CHWs use this information to improve child health programs at the grassroots level. Additional work should be done to explore how CHWs can access the data that they report through mHealth.

## Figures and Tables

**Table 1:** Description of the study validation methods

Implementation strength indicator	Validation method	Description
Validation at the health center/supervisor level		
(1) Percentage of HSAs currently working	Supervisor records	Record review; if no record was available, supervisor responded from memory
(2) Percentage of HSAs trained in CCM	Supervisor records	
(3) Percentage of HSAs supervised in the previous 3 months	Supervisor records and monthly monitoring form	Record review; if no record was available, supervisor responded from memory
(4) Percentage of HSAs who received clinical mentoring in the previous 3 months		
(5) Percentage of HSAs supervised with reinforcement of clinical practice for the most recent supervision in the previous 3 months	Supervision and mentoring checklists	Review of most recent completed checklist; if no checklist was available, information was not captured
(6) Percentage of HSAs with a drug stock-out in the previous 3 reporting months	Monthly monitoring form review	Review of six most recent monthly monitoring forms; if all six were not accessible, then data were recorded from the partial set*
Validation at the village clinic/HSA level		
(8) Percentage of HSAs with current stocks of CCM drugs/supplies	Observation at VC	Direct observation of drugs/supplies at HSA VC
(9) Percentage of HSAs with minimum stocks of CCM drugs		
(10) Percentage of HSAs who have treated a sick child in the previous 3 months	Register review	Direct observation of CCM register at HSA VC

VC = village clinic.

* HSAs were asked only about the stock-outs that had been reported to the health center through the monitoring form so that the information could be validated. It did not include recent stock-outs.

**Table 2:** Implementation strength indicators reported by the HSA versus observed by the interviewers with sensitivity and specificity of the HSA cellphone interview method (weighted)

Implementation strength indicator	Reported percentage (*n*/*N*)	Observed percentage (*n*/*N*)	Sensitivity (%)	Specificity (%)
HSAs working at the time of the assessment	100 (200/200)	100 (200/200)	100	100
HSAs trained in CCM	99 (199/200)	100 (200/200)	99	100
HSAs who received drug box	100 (200/200)	100 (200/200)	100	100
HSAs who have seen a sick child in the past 7 days	89 (178/200)	89 (177/200)	100	97
Average (range) number of children treated in the previous 7 days	11.2 (0–58)	10.8 (0–54)	Bland–Altman mean difference*: −0.365 (CI = −0.939–0.209)	
HSAs supervised in CCM in the last 3 months	30 (60/200)	30 (60/200)	80	91
HSAs mentored in CCM in the last 3 months	29 (57/200)	19 (37/200)	84	84
HSAs supervised with reinforcement of clinical practice for most recent supervision†	41 (82/199)	35 (69/199)	87	83
HSAs with current stocks†				
LA 1 × 6	87 (171/198)	86 (170/198)	100	95
LA 2 × 6	86 (171/198)	87 (172/198)	99	98
Cotrimoxazole	85 (169/198)	85 (168/198)	99	94
ORS	83 (164/198)	82 (163/198)	100	96
Zinc	81 (160/198)	81 (160/198)	100	100
All drugs	60 (118/198)	59 (116/198)	100	98
HSAs with no stock-out in the previous 3 reporting months‡				
LA 1 × 6	87 (78/89)	88 (78/89)	94	59
LA 2 × 6	84 (75/89)	82 (73/89)	94	60
Cotrimoxazole	90 (80/89)	89 (80/89)	100	94
ORS	82 (73/89)	84 (75/89)	97	100
Zinc	80 (71/89)	85 (76/89)	92	90
All drugs	58 (52/89)	65 (58/89)	89	100
HSAs with minimum stocks†				
LA 1 × 6 (six blister packs/36 tablets)	82 (163/199)	82 (163/199)	100	98
LA 2 × 6 (four blister packs/48 tablets)	83 (165/199)	83 (165/199)	100	98
Cotrimoxazole (60 tablets)	83 (165/199)	83 (165/199)	100	100
ORS (12 sachets)	69 (138/199)	70 (139/199)	99	100
Zinc (60 tablets)	67 (133/199)	67 (134/199)	98	98
All drugs	49 (97/199)	50 (99/199)	97	99
HSAs with CCM supplies				
Timer	97 (195/200)	97 (195/200)	100	82
MUAC	96 (192/200)	96 (191/200)	100	91
Sick Child Recording Form†	97 (194/199)	97 (192/199)	99	67
Sick Child†	100 (198/199)	100 (199/199)	100	100

CI = confidence interval; MUAC = mid-upper arm circumference.

* The Bland–Altman test does not take into account the clustered nature of the data because of specifications of the software; however, taking clustering into account tends to increase confidence intervals so that the present non-significant result is unlikely to change had the clustering been taken into account.

† Excluded any HSAs with missing or inconsistent data.

‡ Included only HSAs with a full set of Forms 1A at the health center to perform the validation.
